# Cerebral Perivascular Spaces Visible on Magnetic Resonance Imaging: Development of a Qualitative Rating Scale and its Observer Reliability

**DOI:** 10.1159/000375153

**Published:** 2015-03-19

**Authors:** Gillian M. Potter, Francesca M. Chappell, Zoe Morris, Joanna M. Wardlaw

**Affiliations:** Brain Imaging Research Centre, University of Edinburgh, Edinburgh, UK

**Keywords:** Small vessel disease, Stroke, Leukoaraiosis, Magnetic resonance imaging, Brain infarction, Dilated perivascular spaces

## Abstract

**Background:**

Perivascular spaces (PVS) are an important component of cerebral small vessel disease (SVD), several inflammatory disorders, hypertension and blood-brain barrier breakdown, but are difficult to quantify. A recent international collaboration of SVD experts has highlighted the need for a robust, easy-to-use PVS rating scale for the effective investigation of the diagnostic and prognostic significance of PVS. The purpose of the current study was to develop and extend existing PVS scales to provide a more comprehensive scale for the measurement of PVS in the basal ganglia, centrum semiovale and midbrain, and to test its intra- and inter-rater agreement, assessing reasons for discrepancy.

**Methods:**

We reviewed previously published PVS scales, including site of PVS assessed, rating method, and size and morphological criteria. Retaining key features, we devised a more comprehensive scale in order to improve the reliability of PVS rating. Two neuroradiologists tested the new scale in MRI brain scans of 60 patients from two studies (stroke, ageing population), chosen to represent a full range of PVS, and demonstrating concomitant features of SVD such as lacunes and white matter hyperintensities. We rated basal ganglia, centrum semiovale, and midbrain PVS. Basal ganglia and centrum semiovale PVS were rated 0 (none), 1 (1–10), 2 (11–20), 3 (21–40) and 4 (>40), and midbrain PVS were rated 0 (none visible) or 1 (visible). We calculated kappa statistics for rating, assessed consistency in use of PVS categories (Bhapkar test) and reviewed sources of discrepancy.

**Results:**

Intra- and inter-rater kappa statistics were highest for basal ganglia PVS (range 0.76–0.87 and 0.8–0.9, respectively) than for centrum semiovale PVS (range 0.68–0.75 and 0.61–0.8, respectively) or midbrain PVS (inter-rater range 0.51–0.52). Inter-rater consistency was better for basal ganglia compared to centrum semiovale PVS (Bhapkar statistic 2.49–3.72, compared to 6.79–21.08, respectively). Most inter-rater disagreements were due to very faint PVS, coexisting extensive white matter hyperintensities (WMH) or the presence of lacunes.

**Conclusions:**

We developed a more inclusive and robust visual PVS rating scale allowing rating of all grades of PVS severity on structural brain imaging. The revised PVS rating scale has good observer reliability for basal ganglia and centrum semiovale PVS, best for basal ganglia PVS, and moderate reliability for midbrain PVS. Agreement is influenced by PVS severity and the presence of background features of SVD. The current scale can be used in further studies to assess the clinical implications of PVS.

## Introduction

**Fig. 1:**
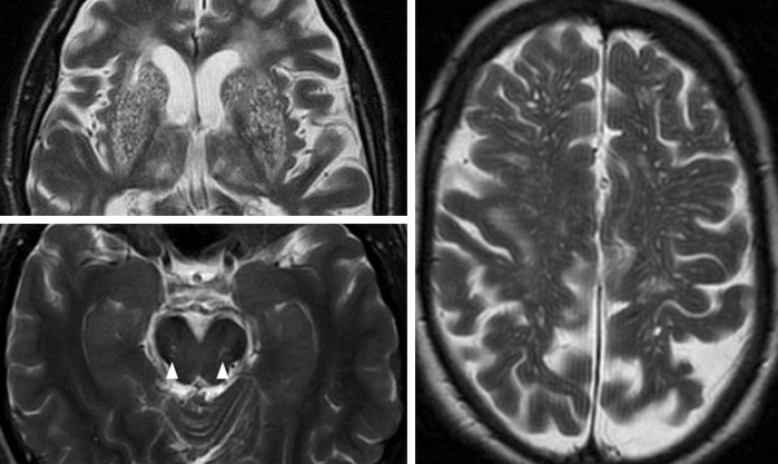
Severe basal ganglia (top left) and centrum semiovale PVS (right). PVS visible in the midbrain (bottom left, arrowheads).

Perivascular spaces (or Virchow-Robin spaces) surround arterioles and venules as they perforate the brain parenchyma [1] and are normally microscopic, but may be visualised on T2- or T1-weighted brain MRI when enlarged (fig. 1). PVS may act as drainage pathways to remove interstitial fluid from the brain [2, 3, 4], so, when enlarged, may represent interstitial fluid trapped in the subpial or interpial spaces [5]. The full diagnostic and prognostic significance of PVS are unknown. PVS appear in small numbers in all age groups, but increase in frequency with advancing age [6, 7], and are associated with worse cognition [8, 9, 10] and with several disease states, including CADASIL (cerebral autosomal dominant arteriopathy with subcortical infarcts and leukoencephalopathy) [11], depression at older ages [12], active multiple sclerosis during inflammation [13], myotonic dystrophy [14], Parkinson's disease [15] and small vessel disease (SVD) in the form of lacunar stroke [16, 17, 18, 19, 20], WMH [18, 20, 21] and vascular dementia [22].

PVS form part of the spectrum of changes seen on MRI in SVD. Until recently, PVS have been somewhat ignored, not being included in any of the existing WMH rating scales [23]. However, a recent international collaboration of experts in SVD has highlighted the need for a robust, easy-to-use PVS rating scale, alongside those in use for WMH and brain microbleeds [24]. Reliable PVS rating scales, whether visual or automated [24] are essential if the diagnostic and prognostic significance of PVS are to be investigated effectively. Several scales have been described (online suppl. table 1; for all online suppl. material, see www.karger.com/doi/10.1159/000375153) [6, 8, 14, 18, 22, 25, 26, 27, 28], but may not adequately capture the range of anatomical coverage, frequency or severity; these have not been extensively tested outside their inception studies.

The purpose of this study was to provide a more comprehensive scale and test its observer agreement, assessing whether raters differed in their assessment of none, mild, moderate, frequent, and severe PVS in the presence of common features of aging and SVD.

## Methods

We considered PVS to be small, sharply delineated structures of cerebrospinal fluid (CSF) intensity measuring <3 mm in cross-sectional diameter, following the course of perforating vessels, round if in axial and longitudinal if cut in the long axis of the perivascular space [24]. We reported the study according to the STRIVE guidelines.

### Development and Extension of Existing PVS Rating Scales

We reviewed previously published scales (online suppl. table 1) [6, 8, 14, 18, 22, 25, 26, 27, 28]. A neuroradiologist applied each scale on two occasions to a test set of 20 standard T2-weighted MR scans from a study of aging, and made incremental modifications to one existing scale by carefully noting benefits and drawbacks of each scale as follows:

### PVS Sites

We retained basal ganglia and centrum semiovale regions (included in all but one of the previously published scales; online suppl. table 1) and added the midbrain (included in one previous scale), as these are three major sites for PVS [1; fig. 1]. We removed the hippocampus location because of its variable visualisation on axial images and because hippocampal PVS may be confused with hippocampal fissural cysts, currently thought to be normal variants. As most people have PVS in the anterior perforated substance [26], we chose not to add this location to the revised scale, for the basal ganglia, as it would not enhance specificity, although this has been included in a recently developed scale, in which PVS are assessed on a single slice at the level of the anterior commissure [28]. We did not separate PVS into sub-regions in the basal ganglia, as we found this difficult and time-consuming [22]. Similarly, we found subgrouping of PVS in the centrum semiovale [25] did not improve intra-rater variability. We tested the effects on intra-rater variability of using predefined slices and predefined regions, for example, for basal ganglia, using the slice showing the maximal area and for centrum semiovale, regions defined by predefined gyri and sulci, for instance, PVS anterior to the central sulcus. None of these improved consistency.

### Method of Rating

**Fig. 2:**
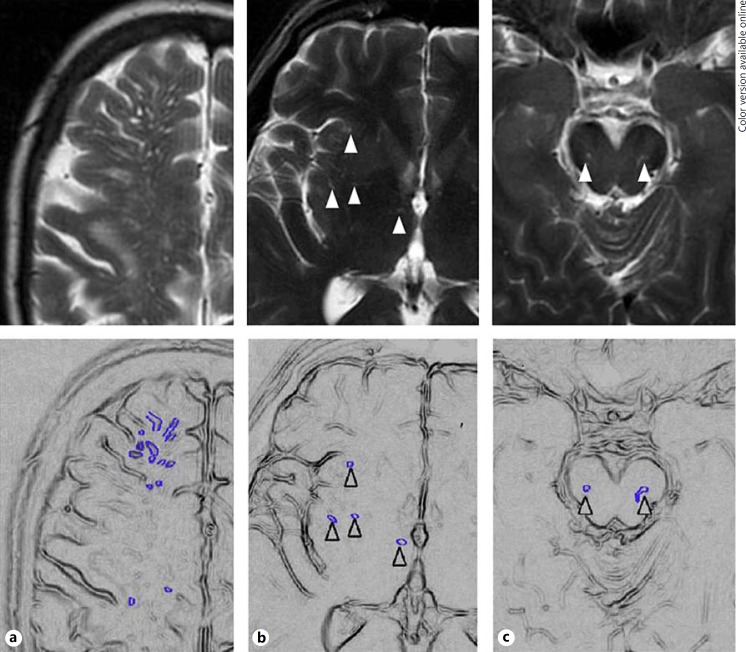
**a** Grade 4 (severe) centrum semiovale PVS (highlighted in schematic). **b** Grade 2 basal ganglia PVS (arrowheads). **c** Visible (grade 1) midbrain PVS (arrowheads).

We retained the frequency and range of PVS used by Mac Lullich et al. [8] as follows: 0 = none, 1 = 1–10, 2 = 11–20, 3 = 21–40, 4 = >40 PVS per region (fig. 2). These categories are similar to those used by Rouhl et al. [25] and seemed to best reflect the potential frequency and range of PVS in a wide range of normal aging and stroke-related disease subjects; some previous scales had rather restricted range and frequency of PVS grades [18, 22, 27] or required every individual PVS to be counted [14], and therefore, this was impractical for use in large studies and reduced reliability.

### Size/Morphology

Unlike two previous studies [14, 27], we did not include size criteria due to difficulties in distinguishing between PVS measuring <2 mm and 2–3 mm on routine images (this could be added in studies with very high-resolution images). We used frequency and range of PVS in preference to morphology. Although excluded in one previous scale [25], we chose to include PVS surrounded by a FLAIR-hyperintense rim, since the exact nature of these is unclear and we saw no reason to exclude them if they met PVS criteria in all other respects.

### PVS Rating Scale User Guide

We constructed a user guide with detailed definitions, descriptions and clear examples (http://www.sbirc.ed.ac.uk/documents/epvs-rating-scale-user-guide.pdf) prior to testing the revised scale, providing examples for each category/region with instructions on slice selection.

### Assessment of Intra- and Inter-Rater Agreement of Revised PVS Rating Scale

Two experienced neuroradiologists (JMW, ZM), blinded to the other's ratings and not involved in the initial testing of published scales or development of the revised scale, tested the modified PVS rating scale by each rating 60 T2-weighted MR scans (with T1-weighted and FLAIR imaging also available), selected from the archives of the Brain Research Imaging Centre to represent a range of PVS, white matter hyperintensities and atrophy, from studies of ageing and minor stroke, on two separate occasions. For the basal ganglia and centrum semiovale PVS were rated from 0 (none), 1 (1–10), 2 (11–20), 3 (21–40), and 4 (>40), using an overall score for both hemispheres by assessing and scoring each hemisphere separately and then using the hemisphere with the higher score where hemispheres were asymmetric. Midbrain PVS were rated 0 (none visible) or 1 (visible). Images were reviewed by each rater in a random order using a web-based random number service (RANDOM.ORG) on each occasion, at least one week apart. MRI scans were chosen to represent a range of few to many PVS from older subjects scanned in research studies on SVD at the Brain Research Imaging Centre and assessed digitally on a high-definition screen using the National Kodak Picture Archiving and Communication System.

### Statistical Analysis

We calculated intra- and inter-observer weighted (quadratic) kappas for agreement beyond that due to chance for the basal ganglia and CS data and simple kappa for the midbrain data. All data were analysed with SAS 9.1 (www.sas.com), R 2.14.2 (cran.r-project.org/), and MH Program 1.2 (www.john-uebersax.com/stat/mh.htm). We also cross-tabulated PVS rating scores and provided the estimates of the overall percentage intra- and inter-rater agreement, calculated by summing the number of times scans were given the same rating and dividing by the total number of scans. We assessed this percentage agreement for the overall score and for basal ganglia, centrum semiovale and midbrain PVS scores.

We also assessed the consistency of rating by whether raters differed in their assessment of PVS severity, both between the first and second scan assessment (intra-rater) and between raters (on the first assessment) using the Bhapkar [29] test of the table obtained by cross-tabulating the scan results.

## Results

### Observer Agreement, Consistency of Rating and Sources of Discrepancy

Overall kappa statistic measurement of agreement for PVS was moderate to good. There was better agreement for basal ganglia PVS than for centrum semiovale PVS, with basal ganglia PVS intra- and inter-rater agreement ranging from 0.76 to 0.87 and from 0.8 to 0.9, compared to 0.68 to 0.75 and 0.61 to 0.8 for centrum semiovale PVS, respectively; table 1. Agreement was lowest for midbrain PVS, with intra-rater agreement ranging from 0.58 to 0.70 and inter-rater agreement ranging from 0.51 to 0.52 (table 1).

Overall, there was more intra-rater consistency (Bhapkar 0.34 to 18.2) than inter-rater consistency (Bhapkar 2.49 to 21.08), and centrum semiovale PVS were rated less consistently than both basal ganglia and midbrain PVS (online supp1. tables 2, 3).

### Percentage Observer Agreement

For all PVS sites, intra-rater percentage agreement was similar to inter-rater agreement, ranging from 0.54 to 0.96 compared to 0.43 to 0.87, respectively (online suppl. table 4). Percentage agreement for basal ganglia and centrum semiovale categories was similar, with intra- and inter-observer agreement for basal ganglia ranging from 0.54 to 0.68 and from 0.65 to 0.77, respectively and for centrum semiovale, from 0.57 to 0.65 and 0.65 to 0.68, respectively. Higher intra- and inter-rater percentage agreement was seen for midbrain PVS compared to both centrum semiovale and basal ganglia PVS (supplementary table 4).

## Discussion

**Fig. 3:**
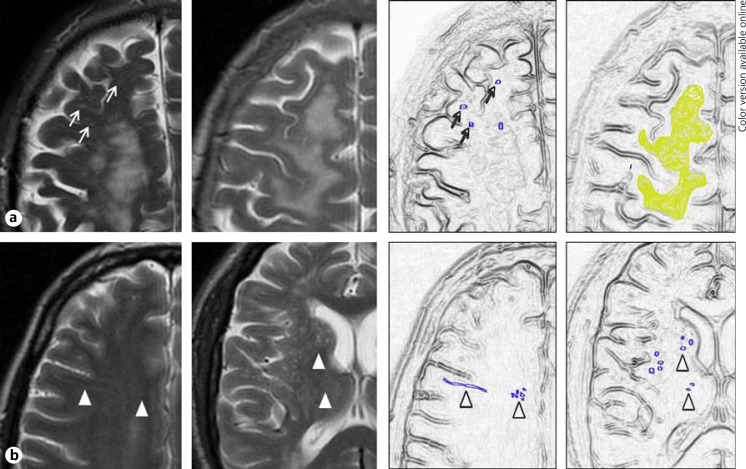
Main causes for observer variability in PVS rating. **a** WMH in the centrum semiovale, with PVS still visible (left) or obscured (right). **b** Small, poorly visualised PVS in the centrum semiovale (left) and basal ganglia (right).

A recent SVD expert collaborative highlights the emerging importance of PVS as an imaging marker for SVD on MRI. We reviewed existing PVS rating scales and developed a more inclusive and robust scale, incorporating all relevant PVS sites and allowing rating of all grades of PVS severity on standard structural brain MR imaging. We found good observer agreement between two experienced neuroradiologists for PVS rating in the centrum semiovale and basal ganglia, and moderate agreement for midbrain PVS. Kappa statistic measurement of agreement and consistency of rating were better for basal ganglia than for centrum semiovale PVS, and lowest for midbrain PVS. Midbrain PVS agreement is likely to be artificially inflated by the limited number of PVS categories to choose from and the fewer number of slices on which midbrain PVS appear. The presence of concomitant WMH and lacunes were the main sources of discrepancy between observers (table 2, figure 3).

The current scale was developed following a comprehensive review of existing PVS scales. We deliberately included MRI scans range of background appearances, including WMH and lacunes, which are more likely to reflect the range of appearances likely to be seen in cohorts of older subjects and stroke patients in whom PVS scales are likely to be used. We included the three main sites where PVS are seen, rather than limiting assessment to restricted brain regions. We assessed PVS on commonly used structural MRI sequences, rather than specialised sequences, helping to increase its general applicability to current research studies of cerebral small vessel disease. We performed extensive, detailed multistatistical analysis of our data.

Our study had limitations. We did not assess atrophy, which may have an influence on PVS severity, but this was not the purpose of this study. The scale was tested by two experienced neuroradiologists, but brain imaging in many research studies is performed by non-radiologists [27] and many raters may be less experienced in reviewing MRI scans and in rating PVS. The use of two raters and experienced raters could have also led to inflated kappa values. Other scales have used multiple observers; however in some cases, the type of rater, and experience in assessing MRI brain images, was unclear; our inclusion of a user guide is designed to help improve observer agreement among observers of difference background and experience. Our revised scale was tested in a cohort of healthy, elderly patients of a similar age and in a stroke cohort; therefore, our results may not be generalisable for all study populations. However, we acknowledge that more studies in larger and different populations are required.

Our finding of lower intra- and inter-rater agreement for centrum semiovale PVS than for basal ganglia PVS confirms previous findings (table 1). Higher overall intra-and inter-observer agreement for all brain regions described by Patankar et al. [22] may reflect the differences in imaging technique or less diseased subjects. We assessed PVS on 5 mm thick contiguous structural brain MR imaging rather than on high-definition 3D sequences, and hence, our data may be more relevant to PVS in other cohorts (table 1). Also high-definition sequences could increase observer disagreement by increasing the visibility of small PVS, which would need to be tested. The overall agreement was higher in all three brain regions in a recently developed PVS scale [28]; however, rating was performed on a single slice for the centrum semiovale and basal ganglia, which, although appearing more accurate, may not be a true or accurate reflection in number or severity of PVS due to individual anatomical variation in the location of PVS, a factor further influenced by differences in patient positioning. Our inter-rater data for basal ganglia PVS are higher than that described by Rouhl et al. [25], with similar results for centrum semiovale PVS, which may reflect the differences in one or several factors, including type of observer, differences in background MRI brain appearances and method of PVS measurement.

Greater variability in rating of centrum semiovale PVS may be partly due to the higher number of potential slices available for rating, with slices containing more or less PVS depending on the level, or to WMH obscuring PVS; in the current study, concurrent WMH, especially when confluent, led to disagreement. PVS may still remain visible ‘through’ WMH. Others have selected only one representative slice for the assessment of number of PVS in the hippocampus, basal ganglia, midbrain and centrum semiovale [28]; this may be oversimplification and problematic given the individual variability in position of PVS in the basal ganglia and centrum semiovale, and in our opinion, the assessment of all slices that include PVS in the brain region being assessed is necessary in order to form an overall impression of severity (or visibility, for midbrain).

At the present time, it is unclear whether increased severity of PVS in a particular brain region has more important clinical implications in the context of cerebral SVD [20]; however, until more data are available, assessment of PVS in each site is likely to be beneficial. Depending on future studies, our scale may require further modification, for example, the removal of centrum semiovale and midbrain locations, with a stronger focus on accurate rating of basal ganglia PVS. The clinical relevance of midbrain PVS is unclear at the present time; however, as this is a frequent site of PVS, it seems relevant to include this region.

Although we have developed and tested a visual rating scale, automated PVS measurement methods may be possible with improved image-processing algorithms in future [30], as has already occurred for WMH rating [31, 32], which may further improve consistency in rating.

The clinical relevance of PVS is currently unclear. A recent international collaboration of SVD experts defining the terminology for imaging features of SVD highlights the need for a robust, easy-to-apply PVS rating scale, which can be used in multicenter research studies. We have developed a scale that enables the rating of PVS to a good level of inter-rater agreement for the two major PVS sites in the basal ganglia and centrum semiovale. This scale should be further tested in different patient populations and by raters with different clinical backgrounds and imaging experience. Studies assessing the pathological role of PVS using such scales will need to consider other components of SVD in multivariable analyses to account for the interrelationships between SVD features.

## Disclosure Statement

None declared.

## Funding

J.M.W. was funded by the Scottish Funding Council SINAPSE Initiative (Scottish Imaging Network, A Platform for Scientific Excellence). G.M.P. and Z.M. were funded by NHS Lothian Research and Development and the Chief Scientist Office of the Scottish Executive. The Lothian Birth Cohort 1936 Study is funded by Age UK and the UK Medical Research Council. The stroke research register was funded by the Wellcome Trust (grant number 063668).

## Supplementary Material

Supplementary data

## Figures and Tables

**Table 1:** Intra- and inter-rater kappa statistics for PVS rating

Sample	Brain region	Rater	Intra-rater kappa (95% CI)	Inter-rater kappa^**^ (95% CI)
Aging study	Centrum semiovale	1	0.68 (0.34–1.00)	0.61 (0.39–0.81)
		2	0.70 (0.49–0.92)	

	Basal ganglia	1	0.80 (0.66–0.94)	0.80 (0.66–0.94)
		2	0.76 (0.35–1.00)	

	Midbrain	1	0.64 (0.01–1.00)	0.51 (0.06–0.96)
		2	0.58 (0.17–1.00)	

Stroke study	Centrum semiovale	1	0.75 (0.62–0.90)	0.80 (0.67–0.93)
		2	0.74 (0.59–0.86)	

	Basal ganglia	1	0.87 (0.79–0.95)	0.90 (0.84–0.97)
		2	0.80 (0.72–0.89)	

	Midbrain	1	0.60 (0.25–0.96)	0.52 (0.21-0.84)
		2	0.79 (0.56–1.00)	

^*^ Calculated using data from first rating.

** Kappa statistics for centrum semiovale and basal ganglia PVS quadratically weighted; quadratic weighting not possible for midbrain PVS due to only two possible categories for PVS rating.

**Table 2:** Causes for intra-rater (**a**) and inter-rater (**b**) disagreement

a		

PVS site	Description	n (%)
Centrum semiovale (n = 20)	Asymmetric/focally dilated PVS	1 (5)
	WMH present^a^	7 (35)
	No WMH present^b^	11 (55)
	Movement artefact	1 (5)

Basal ganglia (n = 8)	Rating 2 versus 3, no clear cause for disagreement	6 (75)
	Unilateral old large cortical infarct	1 (12.5)
	Multiple possible lacunes bilaterally	1 (12.5)

a 1-point different in scale, n = 4; 2-point difference, n = 2; 3-point difference, n = 1. WMH confluent (n = 5) or scattered (n = 2).

b 1-point difference in rating in 10/11 cases.

**Table:** 

b		
PVS site	Description	n (%)
Centrum semiovale (n = 21)	WMH present^a^	9 (42.9)
	No WMH present^b^	11 (52.4)
	Movement artefact	1 (4.8)

Basal ganglia (n = 12)	Rating 2 versus 3, no clear cause for disagreement	5 (41.7)
	Unilateral old large cortical infarct	1 (12.5)
	Multiple possible lacunes^c^	6 (50)

a 1-point different in scale, n = 6; 2-point difference, n = 3; WMH confluent (n = 7) or scattered (n = 2).

b 1-point difference in rating in 10/11 cases.

c Unilateral in 3, bilateral in 3.
