# Supplementary material for: Cerebral Perivascular Spaces Visible on Magnetic Resonance Imaging: Development of a Qualitative Rating Scale and its Observer Reliability
Source: Cerebrovasc Dis. 2015 Mar 19;39(4):224–31. doi: 10.1159/000375153 (PMC4386144; doi:10.1159/000375153)
Supplement: Supplementary file 1 — Supplementary data [file ced-0039-0224-s01.doc]

**Supplementary Table 1.** Summary of existing PVS scales

| Study | Subjects | MRI technique | Locations assessed | Description of rating method | Observer variability |
| --- | --- | --- | --- | --- | --- |
| Adams  2013 [28]  Zhu  2010 [6]  Rouhl 2008 [25] | 65 community-dwellers undergoing MRI, 40 community dwellers >45 years undergoing MRI  1818 community-dwelling patients >65 years  165 patients with first ever lacunar stroke | 1.5T axial T1, T2, PD, FLAIR ; 1.5T axial T1, T2 and FLAIR  1.5T high-resolution T1-weighted brain volume, T2, PD  1.5T axial T2 and FLAIR | CS, BG, hippocampus, mesencephalon  BG, cerebral white matter  CS, BG | Up to maximum of 20 per region; for CS and BG, PVS counted on single, predefined slice for CS and BG  4-level severity score in section containing greatest number  BG: 1=<5, 2=5-10, 3=>10 but still countable, 4=innumerable, resulting in cribriform change  White matter: 1=<10, 2=>10 in total white matter but <10 in section containing greatest number, 3=10-20 in section containing highest number, 4=>20  Low=<20 PVS, moderate=20-50 PVS, high=>50 PVS | Intra-rater ICC: CS 0.88, BG 0.80, hippocampus 0.85, mesencephalon 0.82; inter-rater ICC: CS 0.80, BG 0.62, hippocampus 0.82, mesencephalon 0.75  NA  Interobserver Cohen's kappas: BG-VRs, 0.64; CSO-VRs, 0.57; L-VRs, 0.72 |
| Groeschel 2006 [26] | 125 healthy subjects aged 0.5-30 years; 26 clinical subjects undergoing MRI | Healthy subjects: 1.5T high-resolution 3D structural images using T1W 3D FLASH; clinical subjects: 1.5T, axial T2 TSE/SPIR and FLAIR, sagittal T2, coronal T1 IR-TSE | Supratentorial white matter | Dilated = focal expansion, either irregular or ectatic); non-dilated = smooth and regular morphology | NA |
| Patankar 2005 [22] | 75 patients with Alzheimer's disease; 35 healthy volunteers | 1.5T axial FLAIR, T1W IR, variable echo, FSE, high-resolution 3D T1W fast field-echo | CS, BG, mesencephalon, subinsular region | CS: 0=none, 1=<5 per side, 2->5 on one or both sides; basal ganglia: 0=only either side and <5 either side, 2=>5 SI on either side or any in lentiform nucleus, 2=any in caudate nucleus on either side; subinsular: 0=none, 1=<5 either side, 2=>5 on one or both sides | Intra-rater BG, 0.89-1.00; CS, 0.78/0.82; subinsular, 0.89, 0.91; mesencephalon 0.84, 0.94; interobserver BG 0.91, 0.98; CS 0.84; subinsular 0.90; mesencephalon 0.82 |
| Doubal 2010 [17] | 97 healthy men | 1.9T structural imaging with axial FES T2 | CS, BG, hippocampus | 0=none, 1=<10, 2=11-20, 3=21-40, 4=>40 | Intra-rater Cohen’s kappas: 0.88 for BG; 0.78 for CS |
| Di Costanza 2001 [14] | 41 adults with myotonic dystrophy | 0.5T sagittal T1, axial PD and T2W SE | Lenticulostriate, high convexity | Number multiplied by size category, where size 1=<2mm, 2=2-3mm and 3=>3mm | NA |
| Adachi 2000 [18] | 171 consecutive patients admitted with acute cerebral infarcts | 1.5T T2WI, T1WI and PD | BG | Grade 0, no PVS; grade 1, 1-5 PVS; grade 2, 6-10 PVS; grade 3, >10 PVS | NA |
| Heier 1989  [27] | 816 out-patients undergoing MRI | 3 spin echo sequences (600-800/20; 2000/40-80; sagittal 800/20) | Lenticulostriate, high convexity | 1 (mild)=<2mm, 2 (moderate)=2-3mm, 3 (marked)=>3mm | NA |
| CSF, cerebrospinal fluid; TSE, turbo spin echo; IR, inversion recovery; PD, proton density; SE, spin echo; FSE, fast spin echo; T1W, T1-weighted; T2W, T2-weighted; SPIR, spectral presaturation with inversion recovery; FLASH, fast low-angle shot; CS, centrum semiovale; BG, basal ganglia; FLAIR, fluid-attenuated inversion recovery; CS-O, centrum semiovale oval PVS; L-VR; linear centrum semiovale PVS; SI, substantia innominata; TR, time to repetition; TE, time to echo; CPA, cerebellopontine angle; ICC, intraclass coefficient; NA, Not available | | | | | |

**Supplementary Table 2.** Intra-rater tests for consistency in use of categories between first and second rating, using data from aging (n = 23) and stroke (n = 37) studies. A low p value implies that the ratings were not consistent.

| **Sample** | **Brain region** | **Rater** | **Bhapkar statistic** | **P value** |
| --- | --- | --- | --- | --- |
| Aging study | Centrum semiovale | 1 | 2.19 | 0.53 |
|  |  | 2 | 9.19 | 0.027 |
|  | Basal ganglia | 1 | 3.45 | 0.33 |
|  |  | 2 | NA | NA |
|  | Midbrain | 1 | 1.05 | 0.31 |
|  |  | 2 | 0.34 | 0.56 |
| Stroke study | Centrum semiovale | 1 | 12.48 | 0.0059 |
|  |  | 2 | 13.05 | 0.0045 |
|  | Basal ganglia | 1 | 4.07 | 0.25 |
|  |  | 2 | 18.20 | 0.0004 |
|  | Midbrain | 1 | 0.00 | 1.00 |
|  |  | 2 | 0.34 | 0.56 |

Degrees of freedom for Bhapkar statistic, 3 for centrum semiovale and basal ganglia data, and 1 for midbrain data

NA Not available; the Bhapkar statistic for Rater 2's assessment of the BG between first and second assessment could not be calculated. For the second assessment, Rater 2 did not use Category 4 at all, resulting in a column of zeros in the table cross-tabulating the first and second assessment. It is only possible to calculate the Bhapkar statistic for square tables with at least one non-zero count in every row and every column [Agresti A: Categorical data analysis. 3^rd^ ed. New Jersey: Wiley, 2002: 422.]

Supplementary Table 3. Inter-rater tests for consistency in use of categories at first rating, using aging (n = 23) and stroke (n = 37) study data. A low p value implies that the ratings were not consistent.

| **Sample** | **PVS site** | **Bhapkar statistic** | **P value** |
| --- | --- | --- | --- |
| Aging study | Centrum semiovale | 21.08 | 0.0001 |
|  | Basal ganglia | 3.72 | 0.29 |
|  | Midbrain | 3.45 | 0.063 |
| Stroke study | Centrum semiovale | 6.79 | 0.079 |
|  | Basal ganglia | 2.49 | 0.48 |
|  | Midbrain | 2.87 | 0.090 |

Degrees of freedom for Bhapkar statistic: 3 for centrum semiovale and basal ganglia data, 1 for midbrain data.

**Supplementary Table 4.** Intra- and inter-rater percentage agreement (proportion of scans on which each observer agreed with themselves and with each other) for PVS rating in aging study data (n = 23) and stroke study data (n = 37).

| **Sample** | **PVS site** | **Rater** | **Intra-rater percentage agreement (95% CI)** | **Inter-rater percentage agreement* (95% CI)** |
| --- | --- | --- | --- | --- |
| Aging study | Centrum semiovale | 1 | 0.65 (0.45 to 0.81) | 0.65 (0.45 to 0.81) |
|  |  | 2 | 0.57 (0.37 to 0.74) |
|  | Basal ganglia | 1 | 0.65 (0.45 to 0.81) | 0.65 (0.45 to 0.81) |
|  |  | 2 | 0.61 (0.41 to 0.78) |
|  | Midbrain | 1 | 0.96 (0.79 to 0.99) | 0.87 (0.68 to 0.95) |
|  |  | 2 | 0.87 (0.68 to 0.95) |
| Stroke study | Centrum semiovale | 1 | 0.62 (0.46 to 0.76) | 0.68 (0.51 to 0.80) |
|  |  | 2 | 0.62 (0.47 to 0.76) |
|  | Basal ganglia | 1 | 0.68 (0.51 to 0.80) | 0.77 (0.60 to 0.87) |
|  |  | 2 | 0.54 (0.38 to 0.69) |
|  | Midbrain | 1 | 0.89 (0.75 to 0.96) | 0.84 (0.69 to 0.92) |
|  |  | 2 | 0.92 (0.79 to 0.97) |
